# An ex vivo model for education and training of unilateral cleft lip surgery

**DOI:** 10.1186/s12909-023-04667-6

**Published:** 2023-10-12

**Authors:** Rainer Lutz, Katja Leonie Schulz, Manuel Weber, Manuel Olmos, Tobias Möst, Jan Bürstner, Marco Rainer Kesting

**Affiliations:** Department of Oral and Cranio-Maxillofacial Surgery, University Hospital Erlangen, Friedrich-Alexander-University Erlangen-Nürnberg (FAU), Glückstrasse 11, 91054 Erlangen, Germany

**Keywords:** Unilateral cleft lip, Cheiloplasty, Porcine snout disc, Ex vivo model, Cadaver model, Surgical training, Teaching

## Abstract

**Background:**

Unilateral cleft lip surgery is a complex procedure, and the outcome depends highly on the surgeon’s experience. Digital simulations and low-fidelity models seem inadequate for effective surgical education and training. There are only few realistic models for haptic simulation of cleft surgery, which are all based on synthetic materials that are costly and complex to produce. Hence, they are not fully available to train and educate surgical trainees. This study aims to develop an inexpensive, widely available, high-fidelity, ex vivo model of a unilateral cleft lip using a porcine snout disc.

**Methods:**

A foil template was manufactured combining anatomical landmarks of the porcine snout disc and the anatomical situation of a child with a unilateral cleft. This template was used to create an ex vivo model of a unilateral cleft lip from the snout disc. Millard II technique was applied on the model to proof its suitability. The individual steps of the surgical cleft closure were photo-documented and three-dimensional scans of the model were analysed digitally. Sixteen surgical trainees were instructed to create a unilateral cleft model and perform a unilateral lip plasty. Their self-assessment was evaluated by means of a questionnaire.

**Results:**

The porcine snout disc proved highly suitable to serve as a simulation model for unilateral cleft lip surgery. Millard II technique was successfully performed as we were able to perform all steps of unilateral cleft surgery, including muscle suturing. The developed foil-template is reusable on any porcine snout disc. The creation of the ex vivo model is simple and inexpensive. Self-assessment of the participants showed a strong increase in comprehension and an eagerness to use the model for surgical training.

**Conclusions:**

A porcine snout disc ex vivo model of unilateral cleft lips was developed successfully. It shows many advantages, including a haptic close to human tissue, multiple layers, low cost, and wide and rapid availability. It is therefore very suitable for teaching and training beginners in cleft surgery and subsequently improving surgical skills and knowledge. Further research is needed to finally assess the ex vivo model’s value in different stages of the curriculum of surgical residency.

## Background

The highest standards of patient care, especially for small cleft lip babies, require high-quality training of surgeons. Surgical training and consequently surgical quality can benefit from a simulation-based training approach [1]. Thus, the development of appropriate training models ensures an adequate education of surgical trainees and enhances patient safety. Unilateral cleft lip repair represents a particular challenge in constructive Oral and Cranio-Maxillofacial Surgery (CMF), and the complex region of the lip, nose and upper jaw places high demands on functionality and aesthetics [2]. The surgical goals are to establish form and function, which implies creating muscle continuity of the orbicularis oris muscle, to achieve a satisfactory aesthetic appearance with lip and nose symmetry, to correct lack of vertical length of the cleft-sided philtrum and to create inconspicuous scars [3–5]. The surgeon’s responsibility is high, as patients are young children and inaccuracies can have lasting negative effects on their lives, while an inconspicuous surgical result allows patients to live without disadvantages [6]. Overall, cleft surgery is a challenging field, and beginners in particular should be prepared to invest a significant amount of time and effort in their training. Successful surgery requires a thorough understanding of the anatomical structures and their peculiarities in cleft formation. At the same time there are high technical demands. Beginners may find it challenging to manipulate delicate tissues, and to achieve a good cosmetic result while maintaining proper function. In addition, the variability of cleft types and severity can make it difficult for beginners to know how to approach each case. Cleft surgery is not a one-size-fits-all procedure, and techniques must be adapted to the specific needs of each patient. Indeed, surgical outcomes of cleft surgery are highly dependent on the skills and the experience of the surgeon [7]. Accordingly, there should be numerous opportunities for surgical training in cleft surgery before performing such a procedure on a patient. The benefits of models for simulation training have long since been outlined in other areas of reconstructive oral and maxillofacial surgery [8–10]. Regarding cleft lip interventions, a number of virtual and synthetic-based, high- and low-fidelity simulations have been developed in the recent years, too [2]. Given the complexity of the surgery, low-fidelity models seem inadequate for enhancing one’s surgical comprehension. Virtual simulations, on the other hand, display the surgical steps accurately and increase one’s theoretical knowledge but lack the haptic experience and are therefore unable to build surgical skills and confidence [1]. Haptic high-fidelity models, however, are far from being widely available but are expensive and scarcely acquirable [1, 11]. Moreover, all of them are made of synthetic materials, which do not imitate the properties of real tissue as realistically as cadaver models, that have proven superior in comparative studies [12, 13]. The ideal model enables all surgical steps of a procedure to be carried out as realistically as possible. The goal is to teach the trainees not only cutting and suturing but also how to use the different tissues and rotation- and advancement-techniques to achieve a successful lip closure [1]. Such a realistic training scenario creates situation-specific stress, which can help reduce stress during real operations if successful managed in the simulation [1]. It can be very useful to try the errors and inaccuracies on the models and learn how to correct them instead of avoiding errors at all costs [14]. Because the procedure can be easily repeated on a suitable model, the progress can be continuously assessed by an experienced surgeon to decide when the beginner is able to perform the first surgical steps on the patient or give him more opportunities to practice, make mistakes and learn from them without harming the patient [1, 15]. For this reason, the present study aims to develop an ex vivo model of a unilateral cleft lip, which is of high fidelity, easily and widely available, of low costs and yet able to generate and deepen theoretical knowledge and practical surgical skills.

In accordance with the 3Rs principle (“replace, reduce, refine”; published by Russell and Burch in 1959) for the reduction of animal experiments, only animal slaughterhouse waste with a low utility value was used for the newly developed ex vivo model [16, 17]. The porcine snout disc, which represents the nasal region of the pig, shows many similarities with the human upper lip region in terms of anatomical morphology as well as histology [18]. It therefore fulfils the requirements to resemble the clinical situation adequately and seems to be well suited for further processing to a unilateral cleft lip model, enabling surgeons to practice cleft lip surgery.

## Methods

### Establishing an ex vivo model of a unilateral cleft

**Fig. 1:**
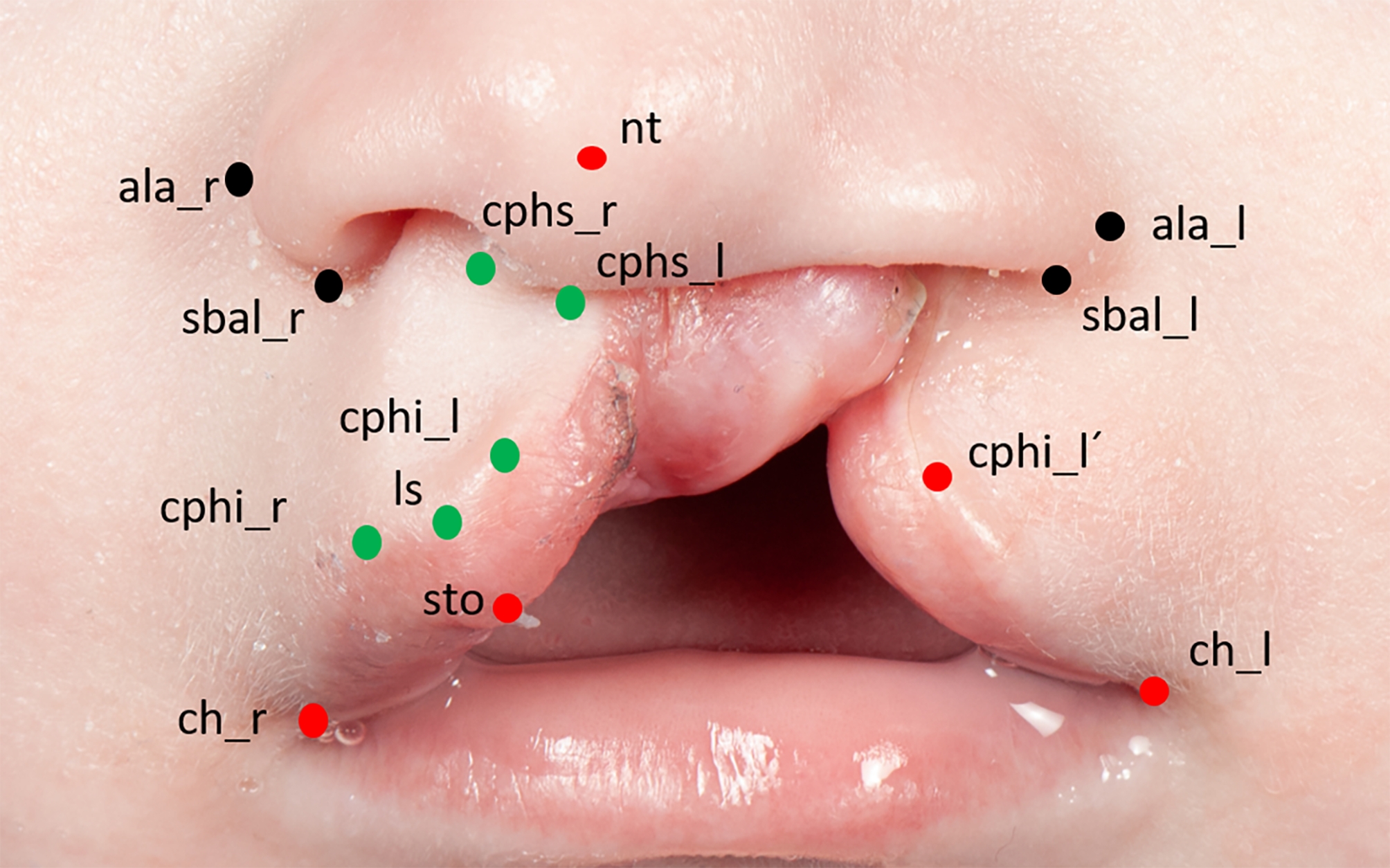
Anatomic reference points on a picture of a cleft patient with unilateral cleft lip and palate (_r = right; _l = left): alare (ala): most lateral point of the nostril; subalare (sbal): most inferior point of the base of the nostril; nasal tip (nt): tip of nose; crista philtri superior (cphs): highest point of philtrum edge; crista philtri inferior (cphi): tip of the cupid’s bow; cphi_l’: cleft sided virtual peak of cupid’s bow; labiale superius (ls): median (lowest point) of cupid’s bow; stomion (sto): lowest median point of upper lip; cheilion (ch): lateral commissure of the lip (right and left)

A child with a left-sided cleft lip and palate from the patient database of our clinic served as a template for the ex vivo cleft lip model. Anatomical reference points were marked on a photograph of the child’s nasolabial area (Fig. 1).

For development of the ex vivo model, pig snouts were provided by a local company (Contifleisch GmbH, Erlangen, Germany). Only fresh snouts (from the day of slaughter) of six months old pigs were used for the study, which were stored in a moist chamber in the refrigerator until use to prevent the skin from drying out. We confirmed sufficient uniformity of the porcine snout discs and defined an appropriate magnification scale prior to the study by analysis of 3D scans of 31 porcine snout discs (data not shown in this study). A photo of the child was printed in the corresponding scale (double magnification) and anatomic landmarks were marked (Fig. 1).

**Fig. 2:**
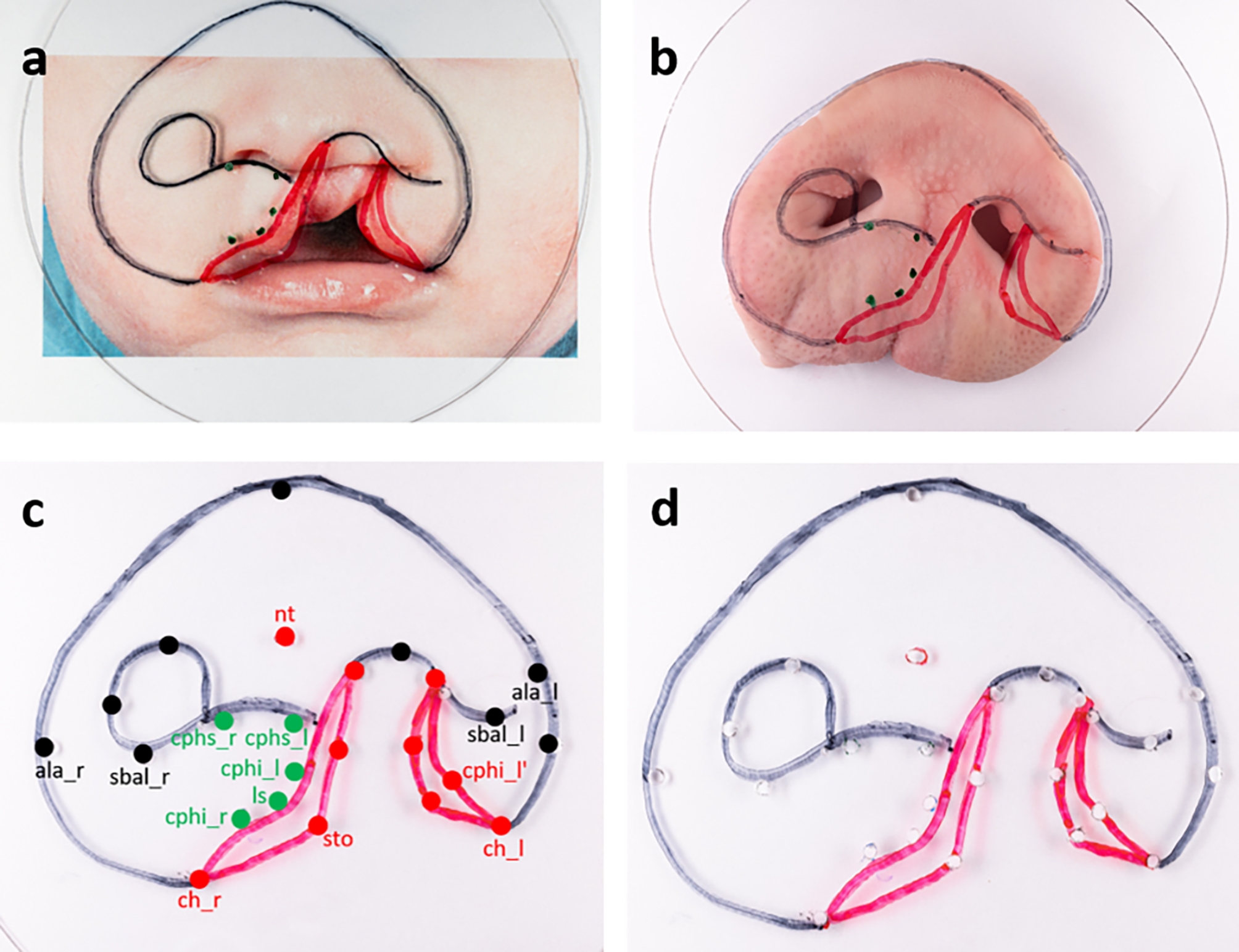
Template foil with anatomic landmarks and reference points of both snout disc and cleft child: on a double magnified picture of the child with a unilateral lip cleft (**a**), on the porcine snout disc (**b**), with labelled reference points (**c**), final template foil with drill holes (**d**). Some points have been digitally repainted to improve visibility

In order to easily and reproducibly generate ex vivo models from the porcine snout discs, we designed a template, using a thermoforming foil made of polyethylene terephthalate (dimension 1.5 × 125 mm, Erkodur, Erkodent Erich Kopp GmbH, Germany) (Fig. 2).

Anatomic contours of an average porcine snout disc were transferred onto the foil with markers (black: EDDING 8404 aerospace marker, edding International GmbH, Germany; red: STAEDTLER permanent Lumocolor, STAEDTLER Mars GmbH & Co. KG, Nuremberg, Germany; green: STAEDTLER non-permanent Lumocolor, STAEDTLER Mars GmbH & Co. KG, Nuremberg, Germany). The same foil was applied to the two times magnified photo of the exemplary cleft child and the anatomic landmarks and reference points of the cleft child’s upper lip and nose region were transferred likewise. To enable transfer of the measuring points to the porcine snout discs, the points on the template were pierced with a drill.

**Fig. 3:**
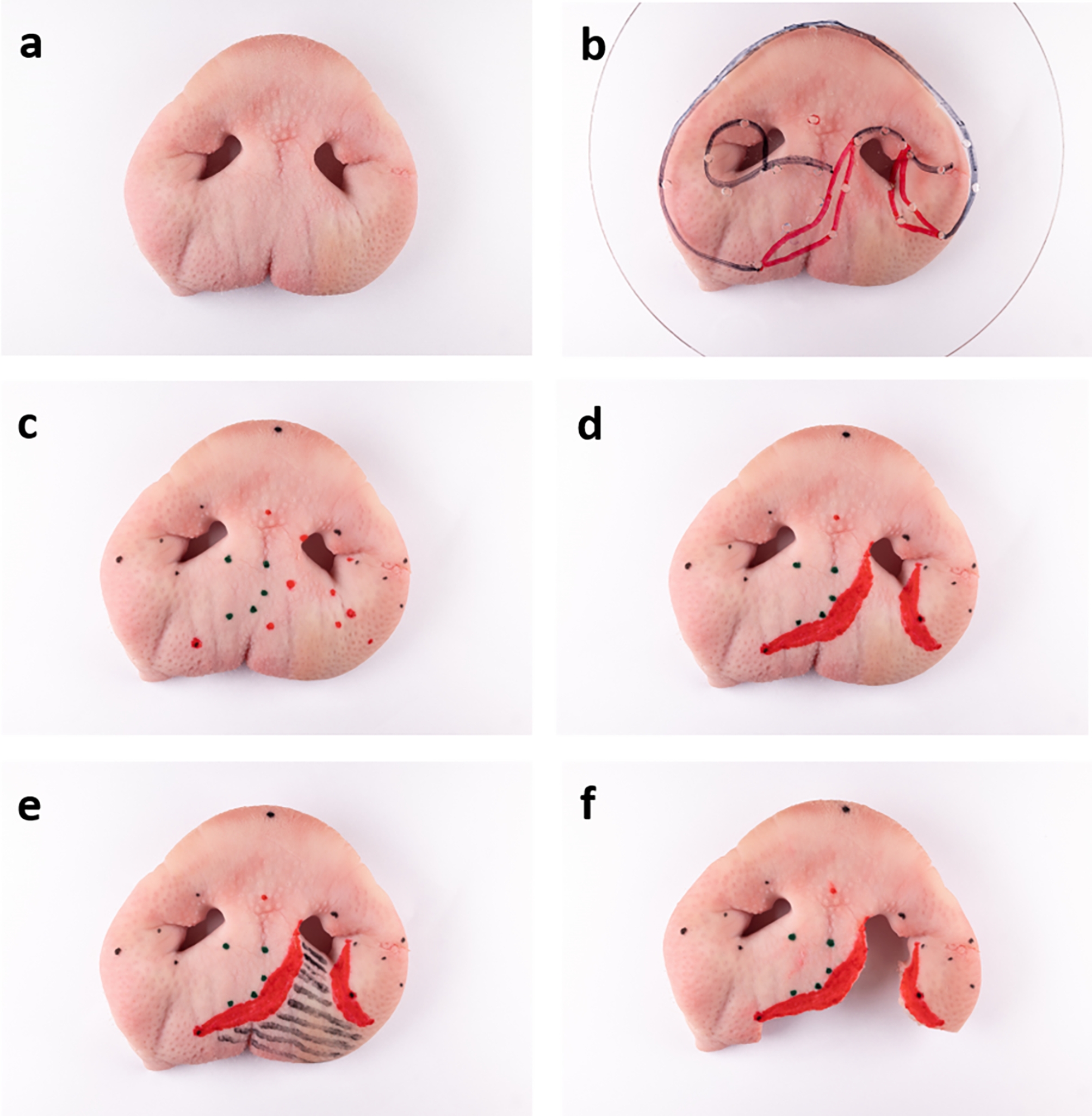
Creation of the unilateral cleft lip model: porcine snout disc (**a**), with template foil (**b**), after marking the anatomic reference points (see Fig. 2c) (**c**), with coloured vermillion (**d**), with shaded cleft area to be excised (**e**), final ex vivo model (**f**). Some points have been digitally repainted to improve visibility

The finalised foil template was now used to prepare the porcine snout disc. Orientated on the indicated landmarks, the foil was positioned on the porcine snout disc. All reference points were marked through the foil template’s drill holes. Subsequently, the vermillion was drawn in with a red permanent marker, and the cleft area was excised to produce the final model (Fig. 3).

### Proof of concept – performing unilateral cleft surgery using Millard II technique on the ex vivo model

**Fig. 4:**
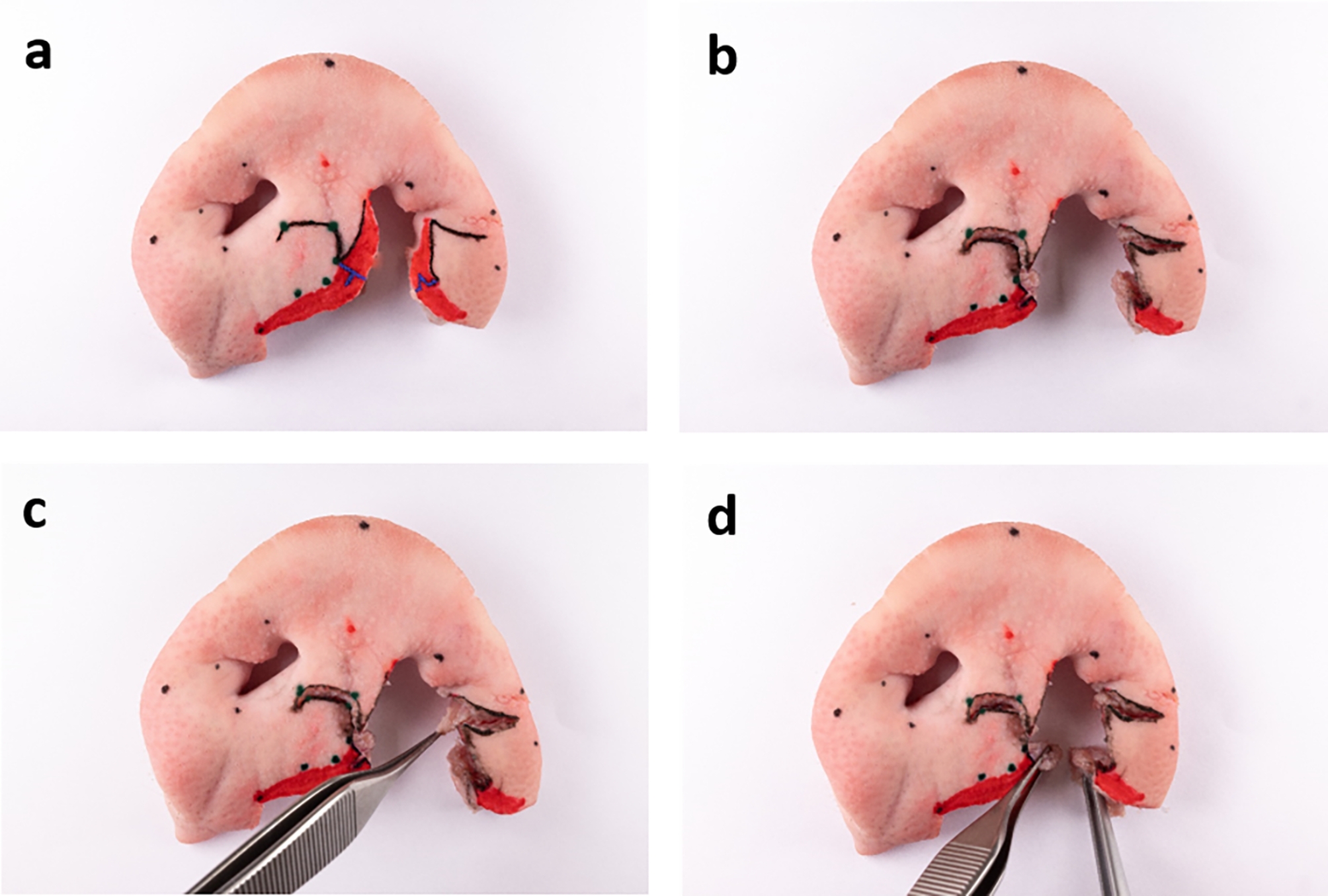
Photo-documentation of Millard II surgery on the ex vivo model, part one: ex vivo model with drawn incision lines according to Millard (black) and Noordhoff (blue) (**a**), after all incisions and the excision of vermillion (**b**), mobilised levator labii superioris alaeque nasi muscle (**c**), mobilised orbicularis oris muscle (**d**). Some points have been digitally repainted to improve visibility

**Fig. 5:**
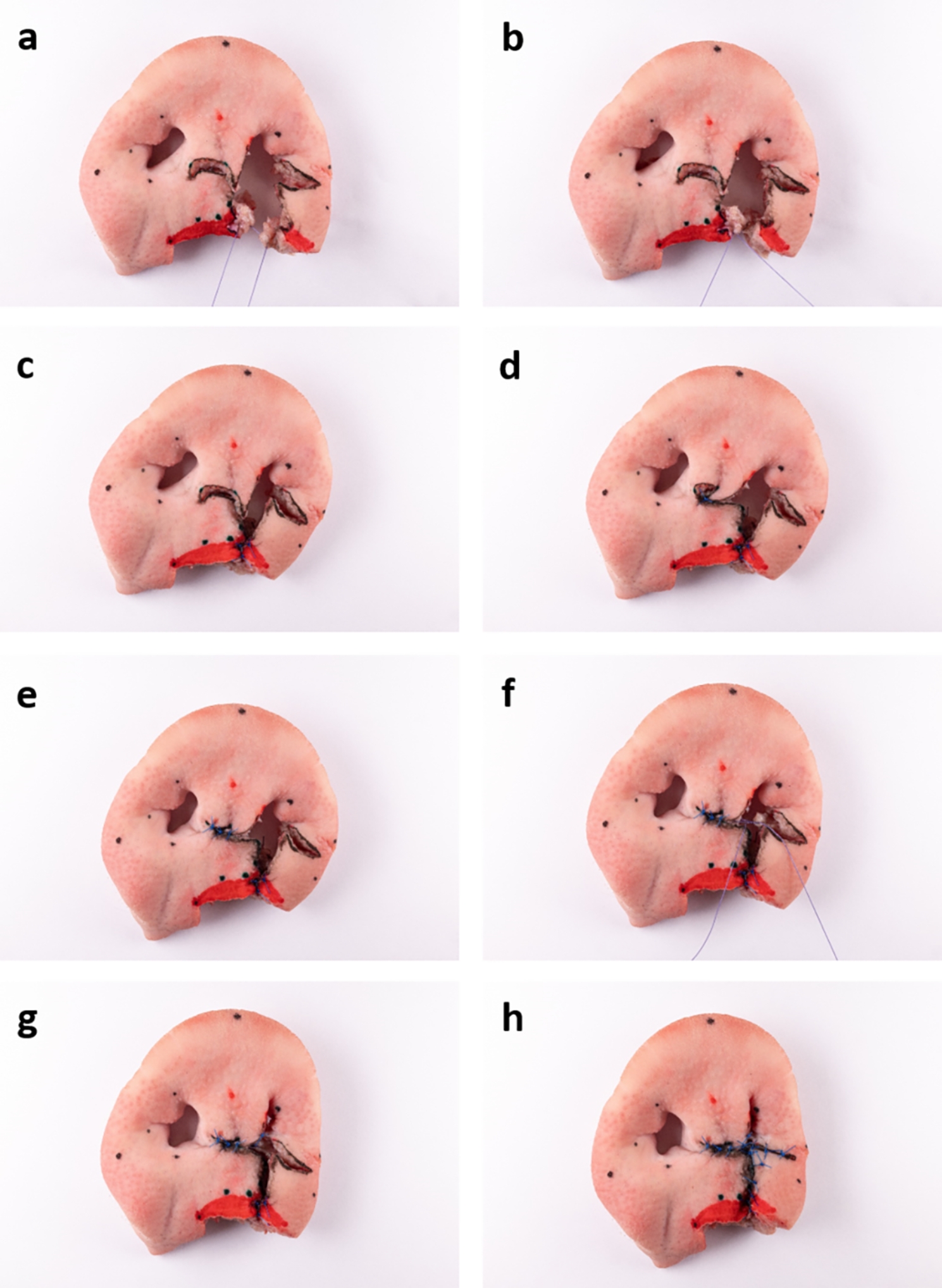
Photo-documentation of Millard II surgery on the ex vivo model, part two: adaption and suture of the orbicularis oris muscle (**a, b**), vermillion plastic according to Noordhoff (**c**), c-flap swung under columella (**d**), c-flap fixated in its new position (**e**), adaption and suture of the levator labii superioris alaeque nasi muscle (**f, g**), final outcome after completed skin closure (**h**)

To test the suitability and practicability of the unilateral ex vivo cleft model for training of established surgical techniques of cheiloplasty, Millard II technique was chosen to be performed on the model. This is (in different modifications) one of the most frequently used techniques for unilateral cleft lip closure [19]. Surgical steps were monitored with the help of photographs (Figs. 4 and 5) and three-dimensional scans (Trios 4, 3Shape, Copenhagen, Denmark). The scanner was validated by the working group in a previous study on cleft models [20].

First, the Millard II incision lines were drawn with a fine permanent marker. Starting on the non-cleft side, a curved line was drawn from point cphi_l to point cphs_l. The line was then continued towards the other nostril, ending at point cphs_r. From this point a “backcut” was drawn caudally. On the cleft side, a line was drawn from the point cphi_l’ along the “white roll” to the nasal entrance. A final line was drawn in an arc below the nostril to the point sbal_l. Noordhoff technique was used for the vermillion procedure. On the non-cleft side, starting from the point cphi_l, a perpendicular line was drawn to the previously drawn line (cphi_l-cphs_l). This line extended to the end of the red of the lips. The incision to accommodate the triangle of the cleft side was placed vertically in the centre of the previously drawn line towards the non-cleft side. On the cleft side, a corresponding triangle was created with its apex in the centre of the vermillion (“wet-dry line”). The short section between the end point of the triangle and the point cphi_l’ of the cleft side was connected with a straight line. Incisions were then traced with a scalpel with an 11 blade and the excess vermilion was cut out. Muscles representing the orbicularis oris muscle as well as the levator labii superioris alaeque nasi muscle were mobilised. Then, the orbicularis oris muscle was reunited and sutured with Vicryl 4 − 0. Next, the vermillion was reshaped by attaching the Noordhoff flaps with Ethilon 5 − 0 and the cphi points of the cleft side (cphi_l; cphi_l’) were fixed to each other at the cranial vermillion border. Then, the c-flap was swung under the columella and fixated. Subsequently, the levator labii superioris alaeque nasi muscle was sewed in order to narrow the cleft-sided nasal entrance. The advancement flap was brought medially and fixed in the desired position with sutures. Finally, the skin was sutured entirely.

**Fig. 6:**
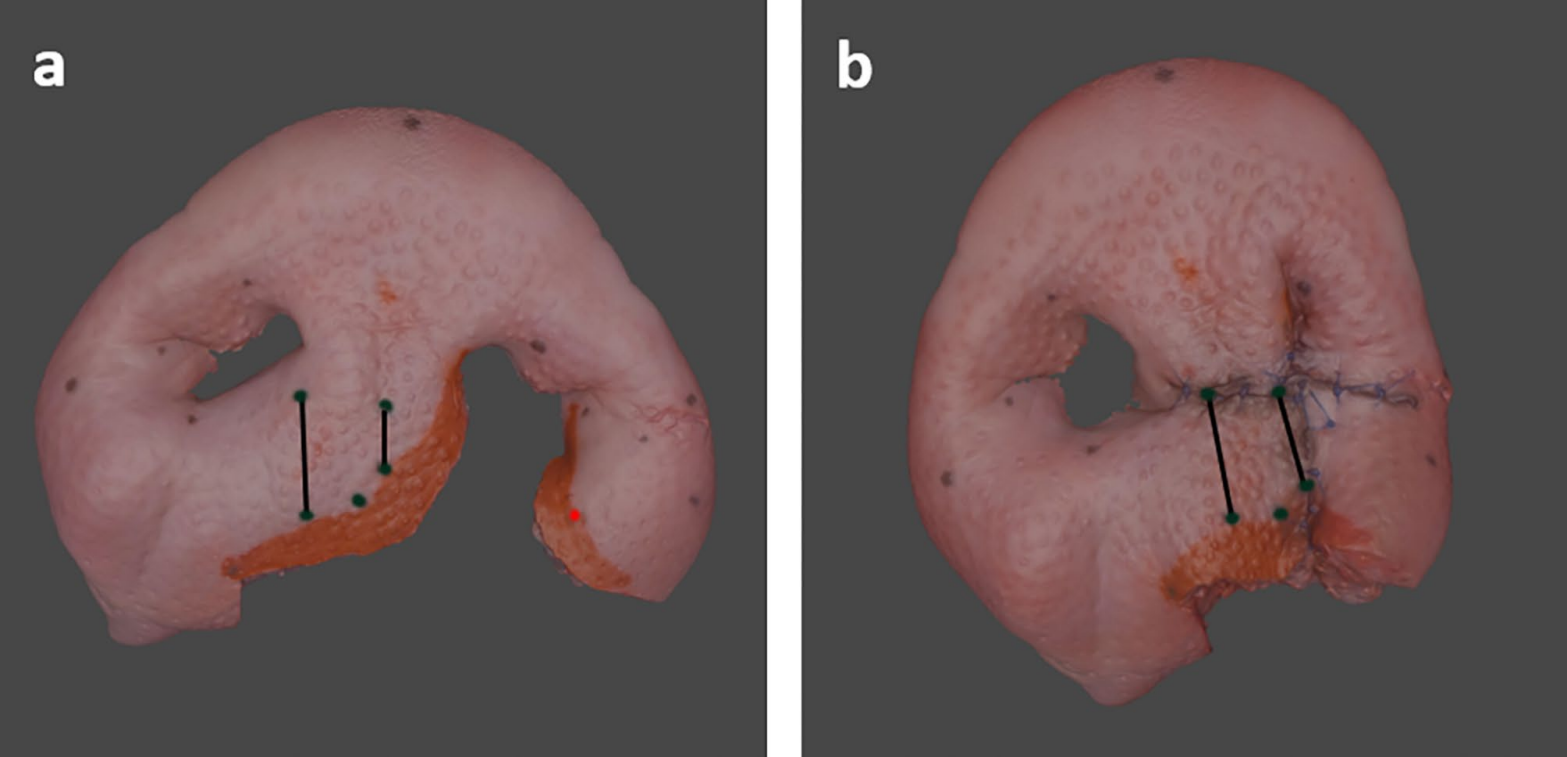
Three-dimensional scans of the ex vivo model before (**a**) and after (**b**) surgery, with marked philtrum edges (black lines). Some points have been digitally repainted to improve visibility

We exemplarily evaluated the final result by measuring the lengths of the constructed philtrum edges on the cleft side and non-cleft side before and after surgery, using the three-dimensional modelling software Blender (Blender Foundation, Amsterdam, Netherlands) (Fig. 6).

### Evaluation

**Fig. 7:**
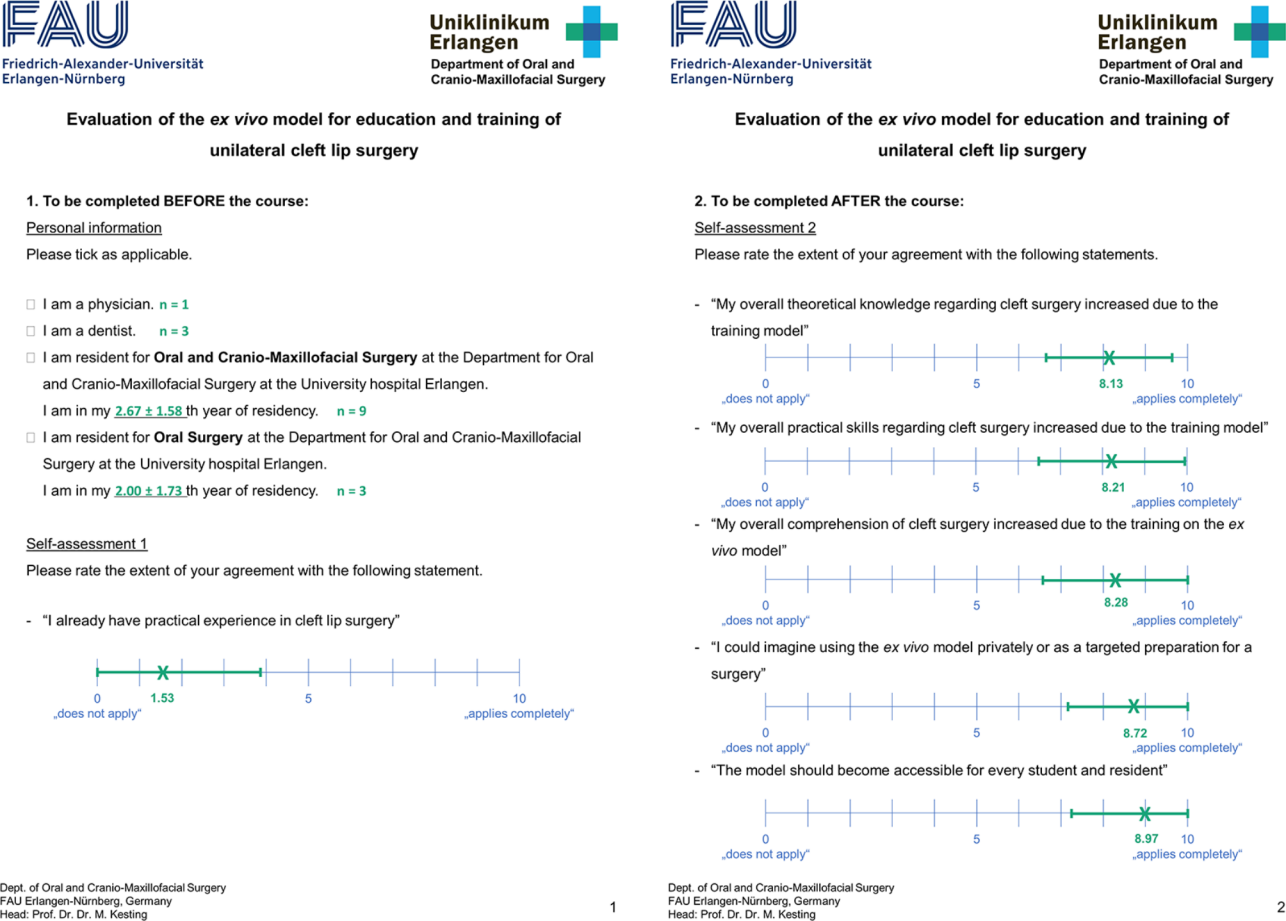
Translated version of the self-designed questionnaire, with the participants’ average answers (X) and standard deviations marked in green

We provided sixteen young medical professionals with a foil template and a porcine snout disc. They received instructions on how to produce the ex vivo model and how to perform treatment according to Millard II and Noordhoff technique. Self-assessment was evaluated using a German questionnaire, which the authors had designed for this study (Fig. 7), with continuous answer scales from zero (“does not apply”) to ten (“applies completely”). A similar questionnaire has been used before by the working group [21]. Before starting, the participants were to give information about their level of experience and indicate whether they “already had practical experience in unilateral cleft surgery”. After training, they were to rate the accuracy of the statements “my overall comprehension of cleft surgery increased due to the training on the ex vivo model”, “my overall theoretical knowledge regarding unilateral cleft surgery increased due to the training model”, “my overall practical skills regarding unilateral cleft surgery increased due to the training model”, “I could imagine using the ex vivo model privately or as a targeted preparation for a surgery” and “the model should become accessible for every student and resident”. The results were evaluated using standard descriptive statistics.

## Results

The porcine snout discs show a very good approximation to a 1:2 magnification of the clinical situation on the cleft child to the ex vivo model. The nostrils on the 1:2 magnified image of the child with the cleft lip correlate very well with the nasal inlets of the pig’s snout. The foil template fitted onto multiple snout discs and is therefore reusable multiple times, simple to use, and causing almost no costs.

Millard II technique was successfully applied on the ex vivo model. The anatomic features represent a very good approximation of the actual surgical area. A multi-layered construction of the lip was simulated realistically in the cleft lip model. By shaping the levator labii superioris alaeque nasi muscle, the cleft sided nasal wing and entrance could be formed. Given that the pig’s nostrils – in contrast to those of a cleft patient – were symmetrical at the beginning, a deliberate asymmetry results after surgery of the model. Thus, the porcine snout, in particular the porcine snout disc, is a suitable model for simulating a unilateral cleft lip after appropriate preparation with the developed template. On the ex vivo model created and ‘treated’ by the authors, the length of the philtrum edge on the non-cleft side was 15.63 mm before surgery and 14.63 mm after surgery. On the cleft side, the philtrum edge measured 8.37 mm before surgery and was extended to 12.35 mm after surgery The difference between the cleft and non-cleft side was thus reduced by 69% (Table 1). Similar values with reduced height of the philtral column on the cleft side and the change in lip height also on the non-cleft side are described for cleft surgery [22–24].

**Table 1:** Measurement of the philtrum edges’ lengths on the ex vivo model before and after surgery

Length of philtrum edge in mm	Absolute value before surgery	Absolute value after surgery	Achieved changes
On the cleft side	8.37	12.35	+ 3.98 (+ 48%)
On the non-cleft side	15.63	14.63	-1.00 (-6%)
Difference between cleft and non-cleft side	-7.26	-2.28	-4.98 (-69%)

**Fig. 8:**
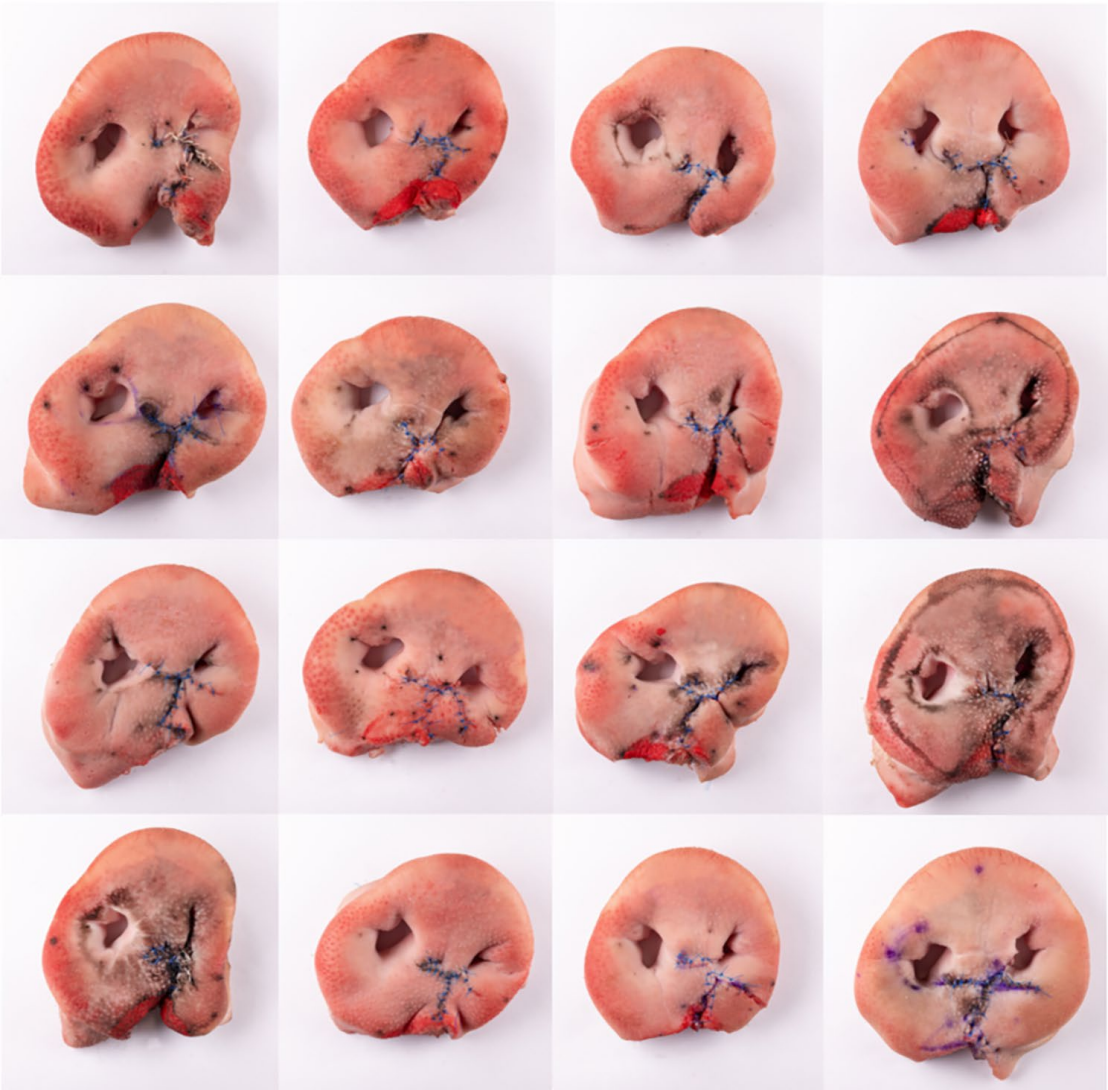
Surgical outcomes of the participants’ Millard II surgeries on the ex vivo model

The outcomes of the participants’ unilateral cleft lip surgery on the porcine snout discs are shown in Fig. 8.

The participants indicated various levels of surgical experience: nine participants were CMF residents in their first to fifth year of residency (average 2.67, SD 1.58), three were residents for Oral Surgery in their first to fourth year (average 2.00, SD 1.73) and four had a medical (n = 1) or dental (n = 3) licence and were interested in a residency in CMF. All participants stated little previous practical experience in unilateral cleft lip surgery (average 1.53, SD 2.33). After their practical training, they indicated a huge increase in their overall comprehension of cleft surgery (average 8.28, SD 1.73), of their theoretical knowledge regarding unilateral cleft surgery (average 8.13, SD 1.49) and of their practical skills regarding unilateral cleft surgery (average 8.21, SD 1.72). All of the participants stated that they could imagine using the model privately or as a targeted preparation for a surgery (average 8.72, SD 1.55) and agreed that the model should become easily accessible to all trainees (average 8.97, SD 1.74). Average values and standard deviations of the participants’ answers are visualized in Fig. 7.

## Discussion

This study aimed to establish a cadaver model as an ex vivo simulation for unilateral cleft lip surgery. In a “proof of concept”, the next step was to investigate whether the model is suitable for performing established techniques of unilateral cleft surgery. In this work, the Millard II technique with Noordhoff vermillion plasty was successfully applied and all aspects of cleft surgery, including muscle plasty, lip plasty and rotation advancement technique, were simulated realistically. The three-dimensional analysis of the final result indicated a clear lengthening of the philtrum edge on the cleft side. Using this method of three-dimensional analysis, even more detailed measurements are possible, e.g. to monitor one’s personal progress after multiple surgical simulations or to compare the outcomes of various surgical techniques or their modifications. Rogers-Vizena et al. evaluated the outcomes of high-fidelity cleft lip simulations with three-dimensional analysis and rated them as a valuable tool for the evaluation of a surgeon’s competence [25]. All participants of the study stated a great learning experience in performing surgery on the model and enhanced their comprehension and theoretical knowledge regarding cleft lip surgery as well as their practical skills. The goal of developing a surgical simulation model for unilateral cleft lip surgery was thus successfully achieved. However, the questionnaire designed for this study is not validated. An objective comparison of the learning experience of the ex vivo model to existing models was neither performed in this study, nor is literature providing adequate data. The long-lasting lack of availability of high-fidelity silicone models made a comparison impossible, at this point, which underlines the urgent need for easily accessible training models. Furthermore, benefits and limitations of the ex vivo model should be assessed with systematic evaluations including surgeons of different levels of experience. Then, the outcomes of the surgical simulations can be analysed in detail to uncover more benefits and limitations, draw conclusions about the cause of inaccuracies and adjust where needed. A final proof of the model’s suitability is only achievable through more research and the limitations in the present study design indicate the next reasonable steps.

According to current literature research (02/2023), the newly developed cadaver model for unilateral cleft lip from the porcine snout disc represents the first ex vivo model for unilateral cleft lip surgery. The availability of realistic models for cleft lip surgery was hitherto mainly limited to virtual or silicone-based training models [11]. A recent review distinguishes between haptic “high fidelity”, haptic “low fidelity” and digital simulation models [2]. Five of these haptic “high fidelity” models have been designed to simulate primary cleft lip surgery. The application goal of these exclusively synthetic-based models was defined as the education and training of cleft surgeons [11, 26]. Although lip and nose symmetry cannot be achieved without adequate rebuilding of the orbicularis oris muscle [27], only the “TORONTO model” [11] and the “HARVARD model” [1] imitate the multi-layering of the tissue. These depict the anatomical conditions - such as skin, musculature and cartilage - in different material components. Yet the high degree of model precision is also reflected in the purchase price - in comparison, the cost of the “TORONTO model” is about $ 250, the “HARVARD model” costs about $ 220 to produce, while the cost estimates for the other models are less than $ 50. In the haptic validation of the “TORONTO model” and the “HARVARD model”, experienced cleft surgeons describe a high agreement with the real surgery with limitations in the area of the nasal structures. However, the synthetic materials used are not able to exactly mimic the properties of real tissue and their overly high form stability impedes an optimal surgical result [25]. Comparative studies have shown superiority of cadaver models compared to synthetic models in similar matters [12, 13]. For this reason, a cadaver model was chosen for surgical simulation in the present study. Due to many similarities in the anatomical morphology of the porcine snout disc and the human upper lip region [18], it was possible to mimic cleft lip anatomy to a large extent. Using cadaver models naturally comes with some disadvantages. The limitations of the ex vivo model lie mainly in the anatomical differences between the porcine and the human upper lip and nose region. Firstly, the pig’s snout disc is flat – therefore, the influence of the cleft surgery on the nasal septum and the nostril cannot be simulated adequately. The intended narrowing of the cleft-sided nostril, though, was clearly observable on the snout disc model und thus successfully imitated. Secondly, the cleft situation is created and therefore muscles are not mis-inserted. The mobilisation of the muscles therefore differs from the real situation. Thirdly, the pig’s epithelium is thicker than a human epithelium [18], making it slightly rougher in its handling, although the differences are less noticeable on the snout disc than on other parts of the porcine skin. On the other hand, this also comes with a higher resilience to minor inaccuracies of the surgeon – especially when compared to synthetic-based models. Its double magnification factor also allows the use of bigger and stronger instruments than usually used for cleft surgery. These facts combined make the porcine snout disc model highly suited to serve as an education and training model for beginners in cleft surgery. When introduced to the subject, it can be hard to immediately understand the complex combination of different techniques for reconstruction of the upper lip. This is where the newly developed ex vivo model can make a valuable contribution as an introduction into the fascinating field of cleft surgery. Even though anatomical variances between the porcine and the human upper lip region exist, the present study proved that this did not constrain the trainees’ great learning experience. Instead, creating the model and marking the anatomical reference points on the snout disc requires transfer thinking, which promotes an implicit learning process. The identification and selection of the adequate points, however, is only two-dimensionally conveyed and only for those who prepare the template foil. The ex vivo model is therefore able to deepen the understanding of beginners in cleft surgery but does not replace the experience made by handling real cleft situations.

The magnification factor of 1:2 is not only beneficial for learning to perform the surgery, but also for analysing the surgical outcomes, since it increases the measuring distances and allows more accurate measurements. It is further advantageous that the snout discs of pigs at a slaughter age of 6 months are relatively uniform in size and model-relevant structure. Therefore, the template developed for the creation of the model is applicable to multiple snout discs, and allows quick, low-cost and easy production of standardised cleft models, while the template itself may also easily be duplicated. Despite its high fidelity, which even allows for multi-layer lip reconstruction, the snout disc model is a very inexpensive cleft model. In contrast to the existing “high fidelity” silicone models, the porcine snout discs can be obtained inexpensively from a butcher or slaughterhouse, as they play only a very minor role in culinary utilisation. The availability is therefore determined by the local amount of pork production and the proximity of the nearest slaughterhouse. The snout discs used in the present work were provided free of charge by the local slaughterhouse. Elsewhere, too, the price per snout disc is likely to be less than one dollar. This makes the unilateral cleft lip training model extremely inexpensive and, combined with the easy and fast manufacturing, paves the way for broad application. The surgical simulation may not only be repeated as often as required for one’s individual training without incurring nearly as much costs as if silicone models were used; the model is just as convenient for teaching many trainees at the same time. To ensure a good teaching quality, group sizes should be adapted to the trainees’ levels of experience, since less experienced trainees need closer supervision. For trainees or students with low levels of surgical experience, the model facilitates the teaching of the idea of surgical lip closure and the corresponding surgical techniques – e.g. rotation advancement technique – in a hands-on setting. Highly experienced surgeons can benefit from the model by repetitive practice as to refine their skills, learning new techniques and even testing new approaches.

In summary, the ex vivo model enables CMF trainees as well as colleagues with different levels of experience to develop surgical skills without expenditure of time or money, and to deeply comprehend cleft surgery by gaining experience on the training model. Thereby, a possible mistake, which must not occur in actual human cleft lip closure, will result in a learning progress of the trainee. The self-assessment conducted in the present study indicated a steep learning curve after only one surgical simulation on the ex vivo model, while the positive feedback from the participants and their responses to the questionnaire emphasised the need and desire for practice opportunities.

## Conclusion

The newly developed ex vivo porcine snout disc model is eminently suitable for simulating unilateral cleft lip surgery. It facilitates CMF residents as well as students and surgeons to learn, comprehend and practice the complex techniques of cleft lip surgery. In contrast to existing models, the ex vivo model combines high fidelity, easy and inexpensive manufacturing and wide availability. This paves the way for versatile application, like using it for gaining experience, for one’s individual surgical training or for the education of larger groups of trainees. Especially CMF residents with basic knowledge in the field of cleft surgery would benefit greatly if the model was included in their surgical training.

## Data Availability

Most of the data generated or analysed during this study are included in this published article. The remaining datasets used and/or analysed during the current study are available from the corresponding author on reasonable request.
